# Evaluation of the National Tips From Former Smokers Campaign: the 2014 Longitudinal Cohort

**DOI:** 10.5888/pcd13.150556

**Published:** 2016-03-24

**Authors:** Linda J. Neff, Deesha Patel, Kevin Davis, William Ridgeway, Paul Shafer, Shanna Cox

**Affiliations:** Author Affiliations: Deesha Patel, Shanna Cox, Centers for Disease Control and Prevention, Atlanta, Georgia; Kevin Davis, William Ridgeway, Paul Shafer, RTI International, Research Triangle Park, North Carolina.

## Abstract

**Introduction:**

Since 2012, the Centers for Disease Control and Prevention has aired a national tobacco education campaign to encourage quitting, Tips From Former Smokers (Tips), which consists of graphic antismoking advertisements that feature former cigarette smokers. We evaluated phase 2 of the 2014 campaign by using a nationally representative longitudinal cohort.

**Methods:**

Cigarette smokers who participated in a baseline survey were re-contacted for follow-up (n = 4,248) approximately 4 months later, immediately after the campaign’s conclusion. The primary outcomes were incidence of a quit attempt in the previous 3 months, intention to quit within 30 days, and intention to quit within 6 months during the postcampaign period. We used multivariate logistic regression models to estimate the odds of each outcome. We also stratified models by race/ethnicity, education, and mental health status. Postcampaign rates of quit attempts, intentions to quit, and sustained quits were also estimated.

**Results:**

Exposure to the campaign was associated with increased odds of a quit attempt in the previous 3 months (OR, 1.17; *P* = .03) among baseline smokers and intentions to quit within the next 6 months (OR, 1.28; *P* = .01) among current smokers at follow-up. The Tips campaign was associated with an estimated 1.83 million additional quit attempts, 1.73 million additional smokers intending to quit within 6 months, and 104,000 sustained quits of at least 6 months.

**Conclusion:**

The Tips campaign continued to have a significant impact on cessation-related behaviors, providing further justification for the continued use of tobacco education campaigns to accelerate progress toward the goal of reducing adult smoking in the United States.

## Introduction

In 2012, the Centers for Disease Control and Prevention (CDC) launched the first federally funded, national tobacco education campaign, Tips From Former Smokers (Tips). Tips consists of graphic antismoking advertisements that feature former cigarette smokers discussing their personal stories of having adverse smoking-related health effects (1,2); the primary goal of the campaign is to encourage smokers to quit. Evaluations of the 2012 Tips campaign showed that it was associated with increases in quit attempts (1), increases in intentions to quit smoking (1), beliefs about smoking-related health risks, concerns about health (3), and increases in nationwide calls to the 1-800-QUIT-NOW quitline portal (4). The 2012 campaign was also cost effective (5). Because of these successes, CDC aired subsequent Tips campaigns in 2013 and 2014. The 2013 campaign aired for 16 weeks from March 4 to June 21, 2013, and the 2014 campaign aired for 9 weeks from February 3 to April 6 (phase 1) and from July 7 to September 7, 2014 (phase 2). Evaluation of the 2013 campaign showed that increased exposure to Tips was associated with increases in quit attempts, especially among African Americans (6).

Although studies suggest that the Tips campaign in 2012 increased quit attempts and intentions to quit, the campaign should be examined over time to determine sustained effectiveness. Phase 2 of the 2014 campaign used a protocol that was similar to that used in previous campaigns, but it lasted only 9 weeks and it had new advertisements featuring new disease conditions. The objective of this study was to assess the effect of phase 2 of the 2014 Tips campaign advertisements on short-term and long-term attempts to quit smoking among a longitudinal cohort of smokers.

## Methods

### Data source

We examined data from online surveys administered to a panel of cigarette smokers in the United States. Survey participants were recruited from a probability sample of postal addresses derived from the US Postal Service’s Deliver Sequence File, which covers approximately 95% of all US households. The survey sample was drawn for our study and did not include participants with known prior participation in survey panels. Survey invitation letters were sent to all sampled households and provided a website link and password to the survey. All surveys were administered online by GFK Custom Research, which recruits and maintains nationally representative online panels.

All analyses for this study were limited to those who were adult smokers at baseline, defined as adults aged 18 years or older who had smoked at least 100 cigarettes in their lifetime and currently smoked either every day or some days at the time of survey. All participants had a known probability of selection and could not volunteer for study enrollment. Participants who did not have Internet access were provided an additional study incentive payment ($20 in addition to a base incentive of $20) to complete the survey in public locations with Internet access, such as libraries. These recruitment procedures are similar to those used in recruitment of GFK’s KnowledgePanel, which are detailed elsewhere (1,7,8). All data used in our analyses were weighted to reflect national distributions of sex, age, race/ethnicity, and education among cigarette smokers from the US Census’ 2010–2011 Tobacco Use Supplement to the Current Population Survey (9).

### Sample

Data were collected before and after phase 2 of the 2014 Tips campaign. The analysis focused on phase 2 because baseline data for phase 1 were not available. Phase 1 of the 2014 campaign included previous campaign advertisements that had been evaluated (1,3,6) and 2 new advertisements that featured Terrie, a former smoker featured in previous campaign advertisements. All smokers who participated at baseline (n = 6,582) were re-contacted for follow-up approximately 4 months later, immediately after the conclusion of the campaign (Figure). The surveys were fielded from April 7 to July 6, 2014 (baseline) and from September 8 to November 17, 2014 (hereinafter, follow-up survey 1). A total of 4,248 smokers completed follow-up survey 1, yielding a longitudinal retention rate of 64.5%. All analyses of the effect of the campaign on quit attempts in this study were based on the longitudinal cohort of 4,248 smokers who completed both baseline and follow-up survey 1, regardless of smoking status at follow-up.

**Figure:**
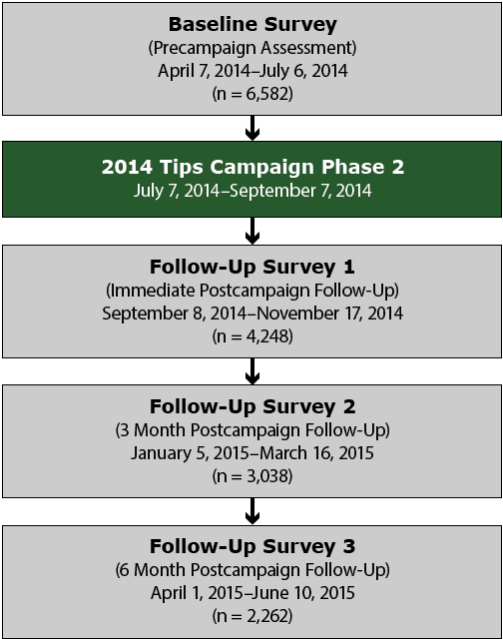
Timeline for phase 2 of 2014 Tips evaluation survey and cohort sample sizes. Sample sizes represent retained cohort sample. In total, 2,262 current smokers and recent quitters participated in all 4 surveys.

After follow-up survey 1, we conducted 2 additional follow-up surveys with this cohort to measure sustained cigarette abstinence among those who reported a quit attempt at follow-up survey 1. These additional follow-up surveys were fielded approximately 3 months (January 5 to March 16, 2015) and 6 months (April 1 to June 10, 2015) after follow-up survey 1 (Figure). A total of 2,262 baseline smokers completed all 4 surveys (34.4% retention from baseline). The analytical sample for estimating sustained quit attempts consisted of the longitudinal cohort of 2,262 baseline smokers who completed all 4 surveys. Unweighted and weighted characteristics of the smoker cohort are shown (Table 1).

**Table 1:** Unweighted and Weighted Sample Characteristics of Smokers at Baseline (N = 4,245), Evaluation of the National Tips From Former Smokers Campaign, 2014–2015

Characteristic	n	Unweighted %	Weighted %
**Age, y**			
18–24	134	3.2	9.8
25–34	370	8.8	19.5
35–54	1,626	38.6	39.2
≥55	2,082	49.4	31.6
**Sex**			
Male	1,776	42.2	53.2
Female	2,436	57.8	46.8
**Race/ethnicity**			
White	3,535	83.9	66.3
African American	305	7.2	14.3
Hispanic	180	4.3	14.1
Other	192	4.6	5.3
**Education**			
Less than high school	293	7.0	15.0
High school graduate	1,120	26.6	43.6
Some college	1,863	44.3	29.3
College degree or more	932	22.2	12.2
**Income, $**			
<20,000	791	19.6	23.4
20,000–49,999	1,504	37.2	36.0
50,000–99,999	1,274	31.5	29.8
≥100,000	470	11.6	10.9
**Health status**			
Has a chronic nonmental health condition	2,981	70.8	60.5
Has a mental health condition	1,388	33.0	34.6

### Measures

#### Outcome variables

To summarize the overall rate of campaign exposure, we measured self-reported recall of advertisements in follow-up survey 1 by using an established protocol for advertisement recognition protocol (1). All respondents were shown, in random order, video streams of each of the 5 campaign advertisements that aired on national television during the campaign. Respondents who were unable to view the advertisements as online video streams were shown a storyboard of images and a script. After viewing each advertisement, respondents were asked to indicate whether they recalled seeing it during the previous 3 months. Hispanic smokers viewed and reported recall of an additional Spanish-language advertisement that aired only on Spanish-language television. Respondents who recalled seeing at least one Tips campaign advertisement were categorized as having exposure to the campaign.

We assessed 3 primary outcome variables at baseline and follow-up survey 1: 1) incidence of a quit attempt in the previous 3 months, 2) intention to quit within 30 days (short-term intention), and 3) intention to quit within 6 months (long-term intention). We measured quit attempt incidence with a dichotomous variable for any quit attempt versus no quit attempt of 1 day or longer. Smokers at baseline who reported nonsmoker status at follow-up survey 1, but reported a quit attempt in the previous 3 months, were also considered to have made a quit attempt. The inclusion of current smokers and recent quitters allowed us to account for total quit attempts among the baseline cohort of smokers. We also created separate dichotomous indicator variables among those who reported smoking at follow-up survey 1 to measure intentions to quit smoking within the next 30 days or within the next 6 months. Sustained cigarette abstinence was defined as the proportion of those reporting quit attempts at follow-up survey 1 who remained abstinent from smoking cigarettes in all 3 follow-up surveys.

#### Independent variables and potential confounders

The primary independent variable was an indicator for the postcampaign period of phase 2 of 2014 Tips, which was used to evaluate campaign-attributable pre–post changes in each outcome variable. We included several additional covariates as potential confounders in the relationship between the postcampaign indicator and the primary outcome variables. Individual-level covariates were sex, age (in years), race/ethnicity, education, annual household income, number of tobacco surveys taken in the previous year, time to first cigarette after waking as a measure of nicotine dependence (in minutes), daily hours of television (in hours), presence of another smoker in the household, presence of a child younger than age 18 years in the household, presence of a chronic (nonmental) health condition, and presence of a mental health condition. We also included covariates for several characteristics of respondents’ media markets, including population size, median income, and proportion of the media market population with a college degree. In addition, we included a market-level variable for gross rating points to account for other Tips advertisements that were aired with state-specific funds during the July to September 2014 time frame of phase 2 of the 2014 Tips campaign. State-level control variables consisted of state per capita tobacco control program funding and cigarette taxes.

#### Statistical analysis

Our analysis began with calculating weighted proportions for campaign advertisement awareness to facilitate estimates of overall campaign exposure among the national population of smokers. We then used multivariate logistic regression models to estimate the odds of each of the 3 primary outcome variables (quit attempts, intention to quit in the next 30 days, and intention to quit in the next 6 months) as a function of postcampaign time, controlling for covariates. All model estimates were weighted and adjusted to account for the clustering of data on individuals (2 observations per person) and the temporal ordering (baseline and follow-up) of observations within each individual data cluster. To examine differences in the magnitude of pre–post changes in outcomes across key subgroups of interest, we estimated additional models stratified by race/ethnicity, education, and mental health status. Two-sided tests at the .05 level were used as our main tests of significance throughout. All analyses were conducted with Stata Version 13 statistical software (StataCorp LP).

Postestimation-predicted values were then used to estimate precampaign and postcampaign rates of quit attempts and intention to quit smoking among the smoker cohort. These rates then were applied to national smoker population totals, derived from 2014 US Census population projections (10) and estimates of smoking prevalence in the United States from the National Health Interview Survey (11). We then estimated the total number of smokers who quit for at least 6 months by applying the sustained cigarette abstinence rate to the estimated total number of smokers who made quit attempts after phase 2 of the 2014 Tips campaign.

## Results

Overall, 79.4% of smokers in our sample reported seeing at least 1 television advertisement from phase 2 of the 2014 Tips campaign. Results from the multivariate logistic regressions (Table 2) show that postcampaign time was associated with increased odds of a quit attempt in the previous 3 months among those who were smokers at baseline (odds ratio [OR], 1.17; *P* = .03) and having intentions to quit smoking within the next 6 months among those who were still smokers at first follow-up (OR, 1.28; *P* = .01). The association between postcampaign time and increased intentions to quit smoking within the next 30 days was not significant (OR, 1.26; *P* = .08).

**Table 2:** Adjusted Odds of Incidence of Previous 3-Month Quit Attempts and 30-Day and 6-Month Intentions to Quit Smoking, by Postcampaign Time, Evaluation of the National Tips From Former Smokers Campaign, 2014–2015

Independent Variables^a^	Incidence of at Least 1 Quit Attempt in Previous 3 Months (Smokers at Baseline)		Intend to Quit Within 30 Days (Smokers at Baseline and Follow-Up)		Intend to Quit Within 6 Months (Smokers at Baseline and Follow-Up)	
	OR (95% CI)	*P* Value	OR (95% CI)	*P* Value	OR (95% CI)	*P* Value
Postcampaign time	1.17 (1.02–1.36)	.03	1.26 (0.98–1.63)	.08	1.28 (1.05–1.55)	.01
Age	0.98 (0.98–0.99)	<.001	1.00 (0.99–1.01)	.66	0.99 (0.99–1.00)	.16
Male	1.03 (0.86–1.23)	.78	0.84 (0.64–1.10)	.20	0.82 (0.67–1.00)	.05
**Education**						
High school graduate	1.28 (0.89–1.84)	.18	1.23 (0.69–2.18)	.49	1.23 (0.83–1.82)	.30
Some college	1.54 (1.07–2.21)	.02	1.92 (1.09–3.36)	.02	2.07 (1.40–3.07)	<.001
College degree or more	1.65 (1.12–2.42)	.01	2.28 (1.28–4.06)	.005	2.35 (1.55–3.57)	<.001
**Race/ethnicity**						
White	0.97 (0.60–1.57)	.89	0.92 (0.51–1.68)	.79	1.06 (0.66–1.70)	.80
African American	2.27 (1.28–4.04)	.005	1.28 (0.63–2.61)	.50	1.86 (1.05–3.28)	.03
Hispanic	1.68 (0.93–3.02)	.08	1.63 (0.78–3.41)	.20	1.52 (0.86–2.69)	.15
**Annual income, $**						
20,000–49,999	0.73 (0.58–0.91)	.006	0.77 (0.52–1.14)	.19	0.75 (0.57–0.99)	.04
50,000–99,999	0.63 (0.50–0.80)	<.001	0.85 (0.58–1.23)	.38	0.89 (0.67–1.19)	.43
≥100,000	0.53 (0.39–0.73)	<.001	0.55 (0.34–0.89)	.02	0.66 (0.46–0.94)	.02
**Other**						
Tobacco surveys taken past year	0.99 (0.87–1.12)	.84	0.91 (0.75–1.12)	.39	0.93 (0.81–1.07)	.31
Media market population size	1.43 (0.71–2.87)	.32	1.43 (0.47–4.38)	.53	0.88 (0.41–1.92)	.75
Median income in media market	1.02 (0.86–1.20)	.86	0.99 (0.80–1.23)	.95	1.08 (0.92–1.28)	.36
% of Media market with college degree	1.00 (0.97–1.04)	.82	1.01 (0.97–1.05)	.63	0.99 (0.96–1.02)	.52
State per capita tobacco control funding	1.00 (0.99–1.01)	.88	1.00 (0.99–1.01)	.96	1.00 (0.99–1.00)	.19
State cigarette tax	1.09 (0.99–1.19)	.08	0.99 (0.87–1.13)	.86	1.02 (0.93–1.11)	.75
Gross ratings points for state-purchased Tips advertisements	1.25 (0.92–1.70)	.15	1.36 (1.20–1.54)	<.001	1.13 (1.03–1.24)	.01
Time to first cigarette, minutes	—b	—b	1.12 (0.72–1.73)	.62	0.95 (0.70–1.30)	.76
Daily hours of television	0.92 (0.87–0.97)	.003	0.95 (0.87–1.03)	.21	0.92 (0.86–0.98)	.01
Other smoker in household	0.86 (0.72–1.03)	.09	0.97 (0.74–1.28)	.83	0.92 (0.76–1.12)	.42
Children in household	1.13 (1.03–1.24)	.01	1.17 (1.03–1.33)	.02	1.10 (1.00–1.22)	.05
Has a chronic nonmental health condition	1.27 (1.06–1.53)	.01	1.36 (1.02–1.82)	.04	1.34 (1.08–1.65)	.008
Has a mental health condition	1.07 (0.89–1.29)	.46	1.11 (0.84–1.48)	.46	1.10 (0.90–1.35)	.34
Model N	7,735	NA	7,132	NA	7,132	NA

Abbreviations: CI, confidence interval; NA, not applicable; OR, odds ratio.

a Age entered into model as a continuous variable. Reference category for race/ethnicity is “other” race; for education, less than high school; for income, less than $20,000.

b Excluded because model included recent quitters (those who were no longer current smokers) at follow-up. This survey item was only asked for respondents at each wave who reported being current smokers.

On the basis of postestimation predictions from these models, we estimated that the population-level quit attempt rate among smokers increased from 37.5% (95% confidence interval [CI], 36.8%–38.2%) before the campaign to 41.9% (95% CI, 41.2%–42.7%) after the campaign (Table 3). In addition, we estimated that the overall proportion of smokers with intentions to quit smoking within the next 6 months increased from 21.1% (95% CI, 20.7%–21.6%) to 25.3% (95% CI, 24.8%–25.9%).

**Table 3:** Predicted Population Changes in Quit Attempts and Intentions to Quit Among Baseline Smokers and Sustained Quits Among Quitters at First Follow-up, Evaluation of the National Tips From Former Smokers Campaign, 2014–2015

Population Outcome	Value^a^
Incidence of past 3-month quit attempt, %	
At baseline	37.5 (36.8–38.2)
At follow-up	41.9 (41.2–42.7)
Increase in population level quit attempts attributable to campaign, in millions, n	1.83 (1.81–1.85)
Sustained quit rate among people who attempt to quit^b^, %	5.7 (3.9–8.2)
Sustained quits attributable to campaign, in thousands, n	104 (103–106)
Incidence of 6-month intention to quit, %	
At baseline	21.1 (20.7–21.6)
At follow-up	25.3 (24.8–25.9)
Population increase in smokers with intention to quit within 6 months, in millions, n	1.73 (1.70–1.77)

a All parenthetical values are 95% confidence intervals.

b Defined as sustaining nonsmoker status at each follow-up survey.

Applying our population-level quit attempt rate to the 2014 US Census population totals, we estimated that phase 2 of the 2014 Tips campaign was associated with approximately 1.83 million additional quit attempts in the United States (Table 3). In addition, we estimated that the number of smokers at first follow-up with intentions to quit smoking within the next 6 months increased by 1.73 million. The estimated 6-month sustained cigarette abstinence rate among those who made quit attempts at first follow-up was 5.7%. Applying this rate to the projected number of additional quit attempts associated with the campaign translated into approximately 104,000 smokers who may have remained abstinent for at least 6 months as a result of the campaign.

Results from the stratified regression models (Table 4) suggest that the postcampaign period was significantly associated with increases in the odds of a quit attempt in the previous 3 months among white smokers (OR, 1.26, *P* = .002) but not among African American (OR, 1.10; *P* = .66) or Hispanic smokers (OR, 0.92; *P* = .76). Some college education was the only educational level associated with higher odds of a quit attempt among smokers (OR, 1.23; *P* = .051). We found a significant association between postcampaign time and 6-month intentions to quit among white smokers (OR, 1.24; *P* = .03) and those with a college degree or more (OR, 1.49; *P* = .04). We also found a significant relationship between postcampaign time and higher odds of a quit attempt among smokers who did not report a mental health condition (OR, 1.24; *P* = .02). Among smokers who did not report a mental health condition, postcampaign time was also associated with intentions to quit within the next 6 months (OR, 1.38; *P* = .02). The campaign was not associated with increased quit attempts or intentions to quit among those who had a mental health condition (OR, 1.10; *P* = .51).

**Table 4:** Stratified Multivariate Logistic Regression Models of Pre–Post Change in Outcomes by Demographic Characteristics, Evaluation of the National Tips From Former Smokers Campaign, 2014–2015

Characteristic	Incidence of at Least 1 Quit Attempt in Previous 3 Months (Smokers at Baseline)		Intend to Quit Within 30 Days (Smokers at Baseline and Follow-Up)		Intend to Quit Within 6 Months (Smokers at Baseline and Follow-Up)	
	OR^a^ (95% CI)	*P* Value (Model n)	OR^a^ (95% CI)	*P* Value (Model n)	OR^a^ (95% CI)	*P* Value (Model n)
**Race/ethnicity**						
White	1.26 (1.09–1.47)	.002 (6,502)	1.16 (0.91–1.49)	.24 (6,004)	1.24 (1.02–1.50)	.03 (6,004)
African American	1.10 (0.73–1.65)	.66 (548)	1.66 (0.71–3.84)	.24 (498)	1.14 (0.64–2.03)	.66 (498)
Hispanic	0.92 (0.55–1.55)	.76 (358)	1.21 (0.51–2.82)	.67 (305)	1.58 (0.67–3.71)	.30 (305)
**Education**						
Less than high school	0.87 (0.58–1.28)	.47 (467)	2.12 (0.73–6.11)	.16 (421)	1.62 (0.80–3.29)	.18 (421)
High school graduate	1.06 (0.83–1.36)	.62 (1,992)	0.97 (0.62–1.52)	.90 (1,832)	1.10 (0.76–1.58)	.61 (1,832)
Some college	1.23 (1.00–1.52)	.051 (3,502)	1.24 (0.89–1.74)	.21 (3,245)	1.25 (0.97–1.61)	.09 (3,245)
College degree or more	1.22 (0.87–1.70)	.24 (1,832)	1.47 (0.82–2.61)	.19 (1,634)	1.49 (1.02–2.19)	.04 (1,634)
**Mental health status**						
Has mental health condition	0.98 (0.77–1.26)	.91 (2,536)	1.09 (0.78–1.52)	.61 (2,349)	1.10 (0.83–1.46)	.51 (2,349)
Does not have mental health condition	1.24 (1.04–1.49)	.02 (5,199]	1.41 (0.96–2.05)	.08 (4,783]	1.38 (1.06–1.80)	.02 (4,783]

Abbreviations: CI, confidence interval; OR, odds ratio.

a Odds ratio indicates the effect of postcampaign time on odds of a quit attempt in model for each characteristic.

## Discussion

Our findings suggest that the Tips campaign continues to have a significant impact on smoking cessation behaviors and intentions to quit smoking among US adult smokers into its third year of implementation. Our results indicate that the quit attempt rate among smokers increased by 17% after the launch of phase 2 of the 2014 campaign. This translates to approximately 1.83 million additional quit attempts associated with the campaign and an additional 1.73 million smokers intending to quit within 6 months after the end of the campaign. Furthermore, phase 2 was associated with an estimated 104,000 6-month sustained quits. These estimates are similar to the results of the 2012 Tips campaign, which estimated an additional 1.64 million quit attempts. The campaign impact persisted even with differences in implementation and baseline conditions: phase 2 of the 2014 campaign was only 9 weeks in duration, whereas the 2012 campaign was 12 weeks in duration, and the baseline previous-3-month-quit attempt rate was higher before the 2014 campaign than it was before the 2012 campaign. These results suggest that the Tips campaign’s effects did not degrade over time, even as the campaign matured and became more ubiquitous, reinforcing previous evidence (1) that brief but high-exposure campaigns can have a significant impact on population-level smoking behaviors.

This study also makes several improvements in the measurement of cessation-related outcomes that enhance the precision of estimated Tips campaign effects. For example, our study captured data on total quit attempts by also assessing quit attempt behavior among baseline smokers who reported being nonsmokers at follow-up. Moreover, long-term follow-up data to estimate the sustained quit rate were collected, whereas previous research (1) relied on extant estimates of a likely sustained 6-month sustained quit rate.

Previous evaluations of the 2012 campaign (1,3,6) relied on existing participants from the online KnowledgePanel. Although this online KnowledgePanel sample is collected by using random address-based sampling methods, it is frequently used for smoking-related surveys because of its large probability-based samples of smokers. Use of the online KnowledgePanel raises concerns that existing KnowledgePanel participants may be more knowledgeable about tobacco-related topics, increasing the potential for survey responses that are not representative of the general smoker population. Our use of a new address-based custom online panel for evaluation of the 2014 Tips campaign eliminates long-term panel conditioning as a likely source of bias. In addition, the similarity of our results to those of the 2012 Tips evaluations (1,3) reduces concerns that the original evaluations were biased by panel conditioning.

Although our findings are similar to previous Tips evaluations for the overall population of smokers, the results of our stratified analysis of minority racial/ethnic groups and educational status differ from the results of previous studies. McAfee et al (1) found an increased impact of the 2012 Tips campaign among African American smokers relative to white smokers and among those with less education (high school or less) relative to those with at least some college. However, these findings were not replicated in our study. One potential explanation for the difference is that the 2014 campaign used a media buy strategy that relied on 2 separate 9-week airings of the campaign that each used different sets of advertisements. We were unable to evaluate the impact of phase 1 of the 2014 campaign; the first phase may have affected outcomes among racial/ethnic and educational subgroups to such a degree that further changes after the second phase were not detectable. In addition, although the study sample permitted the exploration of moderation of campaign effects by subgroups, the sample was not explicitly powered to detect effects in stratified analyses. Because of the variability in sample sizes, any results based on subgroups of the sample should be interpreted cautiously. Alternative study designs with larger samples that are intentionally powered to assess specific subgroups are important to more rigorously evaluate how media campaigns resonate across vulnerable populations that may have a higher burden of tobacco use or tobacco-related diseases.

Our study is subject to at least 4 limitations. First, although we recruited a new custom online panel dedicated only to this study, those agreeing to participate in an online panel may not be representative of the general population and therefore may underrepresent some groups such as rural smokers. Second, we were not able to evaluate the impact of phase 1 of the 2014 campaign because we did not have data before implementation; however, no new creative content was used for the phase 1 campaign, and all advertisements were from prior campaign years. Therefore, our evaluation of phase 2 of 2014 Tips would be more comparable to the 2012 Tips evaluation, where all advertisements were new. Third, although we measured pre–post changes in quit attempt rates for phase 2 of the 2014 campaign, there may have been a latency effect from phase 1 of the 2014 campaign, as well as previous campaign years, which may explain the higher incidence of quit attempts at baseline for 2014 Tips compared with baseline for 2012 Tips. Finally, the 2015 Tips campaign was on the air when 6-month follow-up data on sustained quits were collected, potentially modifying sustained cessation behavior during this period. However, our estimated sustained 6-month quit rate was very similar to the literature-derived sustained quit rate used in the 2012 Tips evaluation (1).

Two years after the first federally funded, nationwide Tips campaign was first introduced, we found evidence of its continued and significant impact on cessation-related behaviors, including an increase in quit attempts and sustained quits as well as an increase in intention to quit within 6 months. This finding has important implications for the value of sustained, long-term tobacco education campaigns. Despite the significant investment required to implement campaigns of this scale, our results suggest that these campaigns continue to have significant impact, even after multiyear implementations. The ongoing health and economic burden posed by cigarette smoking indicates a role for expansion of evidence-based initiatives. These data provide further justification for the continued use of tobacco education campaigns by federal and state health agencies to accelerate progress toward the goal of reducing adult smoking in the United States.
